# Supplementary figures and images for: Phylogenomic analysis and revised classification of atypoid mygalomorph spiders (Araneae, Mygalomorphae), with notes on arachnid ultraconserved element loci
Source: PeerJ. 2019 May 3;7:e6864. doi: 10.7717/peerj.6864 (PMC6501763; doi:10.7717/peerj.6864)

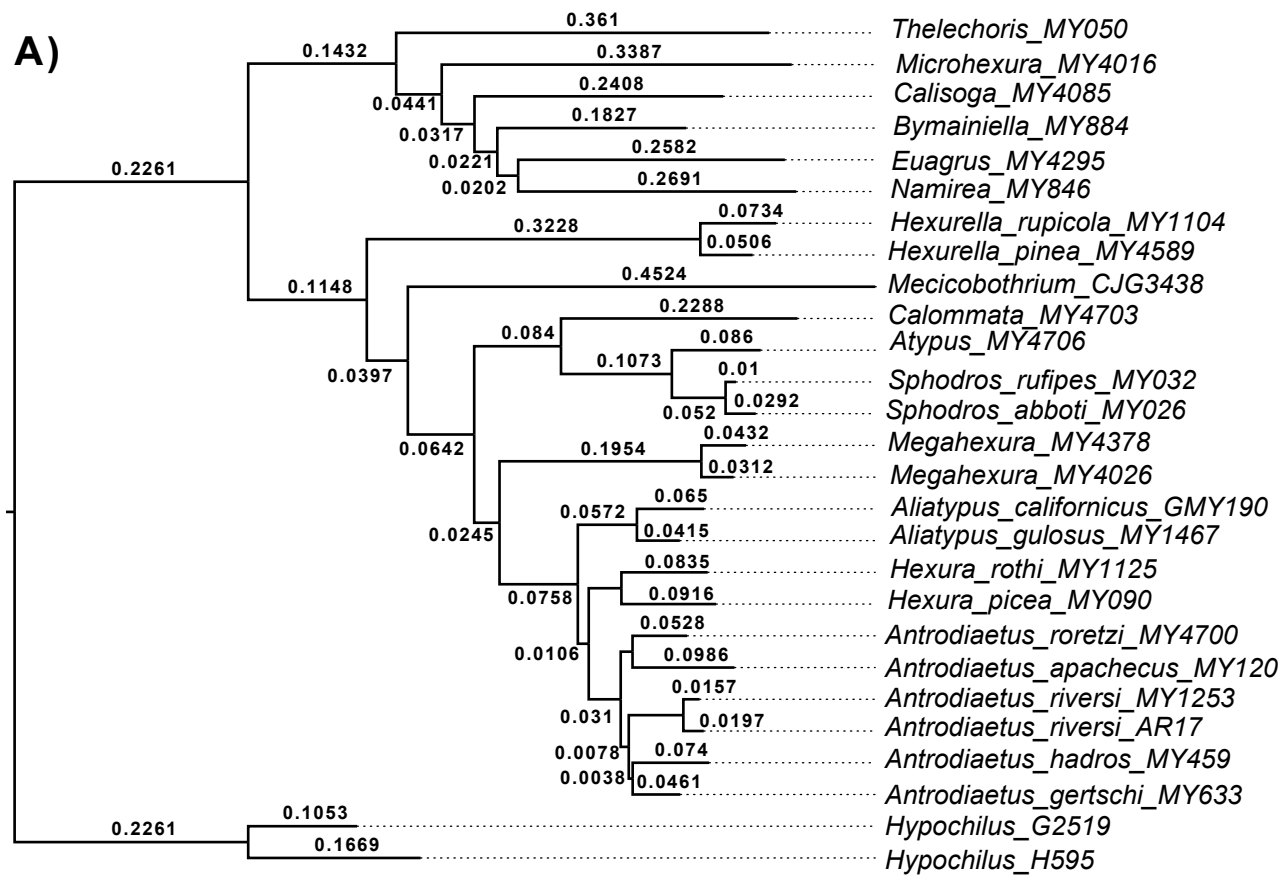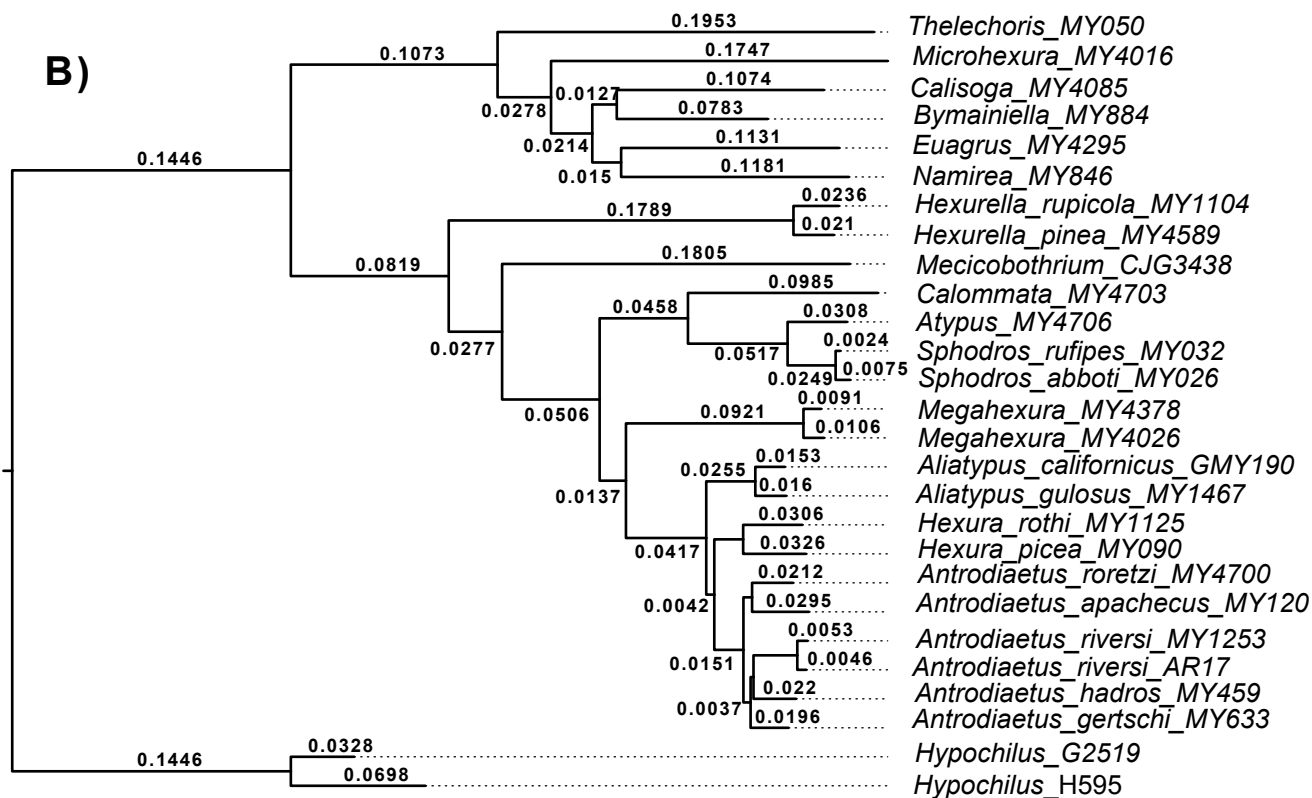

Supplement: Figure S1 — (A) Phyluce unfiltered and (B) “Filtered 70% Exon + Intron” matrices. [file peerj-07-6864-s004.pdf]

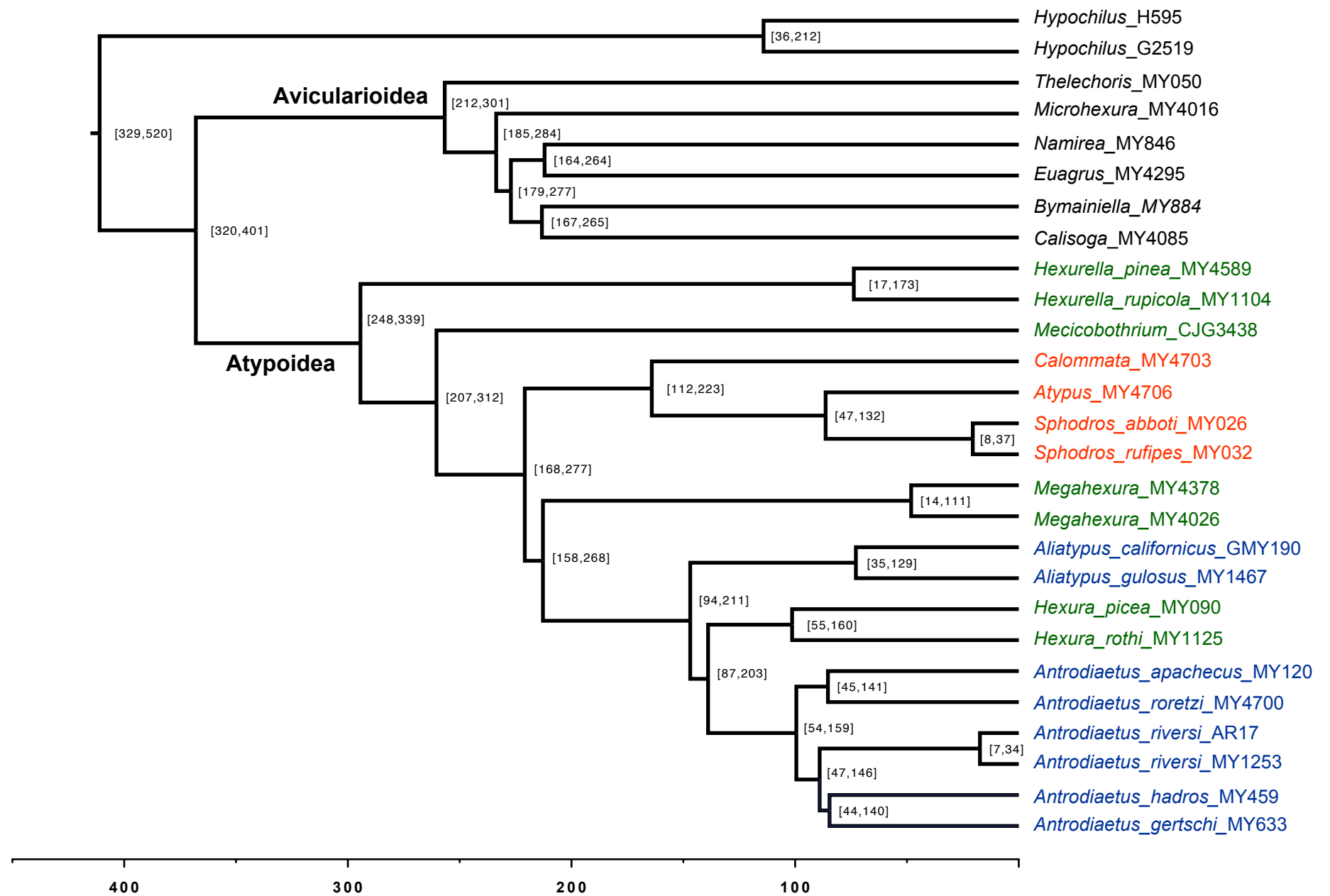

Supplement: Figure S2 — Estimated using calibration with minimum age for the root node of common ancestor of Atypidae < > Antrodiaetidae at 100 MYA, as described in text. [file peerj-07-6864-s005.pdf]
